# Work-Related Musculoskeletal Disorders among Office Workers in Higher Education Institutions: A Cross-Sectional Study

**DOI:** 10.4314/ejhs.v30i5.10

**Published:** 2020-09

**Authors:** Obinna Chinedu Okezue, Toochukwu Henry Anamezie, John Jeneviv Nene, John Davidson Okwudili

**Affiliations:** 1 Department of Medical Rehabilitation, Faculty of Health Sciences and Technology, University of Nigeria Enugu Campus, Nigeria; 2 Department of Physiotherapy, Alex Ekwueme Federal University Teaching Hospital, Abakaliki, Nigeria

**Keywords:** Work-related Musculoskeletal Disorder, WMSD, Office workers, Higher education institution, HEI

## Abstract

**Background:**

Work-related musculoskeletal disorders (WMSDs) currently pose a challenge to public health and elicit considerable financial, physical and social problems for workers. There is a need to attain a deeper understanding of this predicament among office workers, in order to tackle it successfully. This study sought to investigate the prevalence of WMSDs among office workers in Higher Education Institutions (HEIs) as well as discover its associations with their personal/work details and reported risk factors.

**Methods:**

A cross-sectional survey was executed among 217 office workers in different HEIs, who filled self-report questionnaires on WMSDs. Data were analyzed via descriptive and inferential statistics.

**Results:**

The overall prevalence rate of WMSDs was 71.9% among these staff. The lower back, wrists/hands and shoulders were the most reported body regions for these disorders. WMSD prevalence had significant associations with sex (p = 0.004), age (p = 0.028), working hours (p = 0.003) and work experience (p = 0.014). There were significant positive relationships (p < 0.05) between WMSD prevalence and these risk factors: awkward posture, sustained body position, improper bending, workplace stress, inappropriate furniture and inadequate rest breaks.

**Conclusion:**

Most of the study participants were affected with WMSDs, which were primarily reported in the back and upper extremities. Office workers who were older, female, more experienced and work for longer hours, displayed higher risks for these disorders and should be given special attention. Several factors reported by these HEI staff were revealed to significantly influence WMSD prevalence, emphasizing the need for their effective detection and curtailment.

## Introduction

Globally, individuals are now living longer with the consequence of chronic non-communicable diseases and injuries especially musculoskeletal disorders (1). These disorders are presently the most common work-related health problems and the leading cause of health-related absence from work (2). Pain, muscle tightness, joint stiffness and swelling in the affected areas have been observed alongside other symptoms in persons with work-related musculoskeletal disorders (WMSDs), and these could ultimately elicit a disability or career ending injury (3). Hence, there is a rapidly growing universal body of knowledge and attention given to WMSDs, as they pose a major challenge to public health and are an economic burden to health insurance schemes, employers and workers (4).

Systematic reviews on WMSDs in respective occupations across several countries have provided distinct prevalence rates, body distributions and risk factors. Das et al. revealed that handicraft workers were highly vulnerable to WMSDs with prevalence rates of 38.5% – 100%, and identified the neck, back, knees and upper limbs as the most affected body areas (5). WMSD prevalence in the catering industry was observed to be wide-ranging, from 3% – 86%, which was influenced by the difference in catering outfits and job positions (6). In the healthcare sector, up to 90% of physiotherapists have WMSDs during their careers, and these mostly occur at the lower back (7) while about 71.85% of nurses experienced these disorders which primarily presented at the lower back, neck and shoulders (8). Seventy-seven percent of farmers were observed to have WMSDs, commonly at the lower back per year (9) as most construction workers (51.1%) were similarly noted to have these disorders in this body region (10). Key personal/work risk factors associated with WMSD prevalence were job demands (6,8), age and sex (5,7) while awkward postures, excessive repetition and heavy lifting were notable biomechanical risk factors revealed by another review that assessed diverse occupations (11).

Evidence from aforementioned reviews highlight that different employees encounter unique and contemporary challenges which could elicit WMSDs. Office workers who play invaluable roles in various organizations including higher education institutions (HEIs), have also been reported to be affected by WMSDs in several countries (11,12). Specific research on office workers show a general high WMSD prevalence, as all the following literature reported at least one disorder at any point in time. Chinese office workers were observed to primarily present with WMSDs at the neck region (13), and this trend was noted in their Thai counterparts (14). Studies in Turkey, Brazil, Iran and Kuwait showed that WMSDs mostly occurred at the lower back stating rates above 51% (15–18), with a study accentuating that awkward postures posed significant risks for WMSDs (15). Furthermore, age (14,18,19) and sex (14,18–20) have also been revealed as key risk factors for these disorders among office personnel.

The problem of WMSDs among office workers extends to Nigeria, as some studies have shown significant prevalence rates among those in the civil service (21–23). Other surveys among local HEI staff revealed a high WMSD prevalence (above 60%) in groups comprising office workers (24,25). Particularly, about 70% of Nigerian HEI office workers experienced these disorders which mostly occurred at the lower back, and were reported to be associated with their sex (26) and employment duration (27). However, there is limited information on WMSDs primarily regarding the diverse risk factors peculiar to local office staff. Considering the above deficit with the stated high WMSD prevalence and current proliferation of HEIs in Nigeria, the need to obtain detailed data on this population arises. This would enhance a deeper understanding of the predicament and help in the successful development of interventions or strategies to check these disorders. Hence, this study sought to investigate the prevalence of WMSDs among office workers in different HEIs and discover its associations with their personal/work details and reported risk factors.

## Materials and Methods

**Research design**: A descriptive cross-sectional design was utilized for this study. Participants were recruited through purposive sampling in 4 prominent HEIs in Enugu State, Nigeria. Nonacademic employees, who worked in various offices across the different departments/units of the aforementioned institutions, were identified from their respective staff registers and screened to ascertain their eligibility to participate. Ethical clearance was obtained from the Health Research Ethics Committee, and permission got from the various heads of departments/units in each educational institution, before commencing the study.

**Participants**: A total of 217 (out of 260 recruited) office workers, between 21 and 60 years, involving 91 males and 126 females participated in the study. Participants were selected if they solely performed administrative duties and were designated as secretaries, clerks or typists. Staff who were newly employed (less than a year), pregnant or had chronic systemic illness were excluded alongside those with recent surgeries or fractures. The employees were informed about the study objectives and the data collection process, assuring anonymous and voluntary involvement. Informed consent was obtained accordingly from all participants.

**Instrument**: A specially designed self-report questionnaire comprising three sections was used for this study. Section 1 collected the participants' personal and work details: age, sex, marital and educational statuses as well as institution, work experience and working hours per day. Section 2 considered the prevalence of WMSDs among the participants. The standardized Nordic Questionnaire developed by Kuorinka et al (28) was incorporated and used to assess the 12 months' prevalence of WMSDs with their distribution across various parts of the body. This tool has been extensively used (29) and reported to possess good psychometric properties (30). Section 3 collected data on the participants' report of relevant risk factors. Ten pertinent factors that had been stated in previous literature (11,15,25,27) were considered and adopted to form a scale to meet this objective. Participants identified the relevance of each factor towards their predisposition to/attainment of WMSDs rated on a four-point scale, from ‘strongly agree’ to ‘strongly disagree’. In the study sample, this scale was observed to be internally consistent with Cronbach's alpha value of 0.82.

**Procedure**: The study instrument was scrutinized by 6 health professionals who corrected typographical errors and subsequently, agreed that it was properly constructed to achieve the objectives of the study. A pilot study involving 8 eligible office workers was conducted, and this yielded some positive feedback as the participants revealed that the instrument was clear, easy to understand and complete. These participants were later interviewed and their verbal responses matched those they had filled in the questionnaire, revealing its reliability. Duplicates were then given to 260 office workers, and only 217 questionnaires were returned to the researchers, thus depicting a response rate of 83.5%. Information was scrupulously extracted from the forms, which were all filled correctly, and kept confidential.

**Data analysis**: SPSS Software version 23 for Windows was used to analyze data. The participants' details, prevalence and body distribution of WMSDs were presented descriptively by using percentage and frequencies. Association between the prevalence of WMSDs and participants' details was examined using the Pearson's chi-square test. Spearman's rank correlation was used to assess the relationship between the reported risk factors and prevalence of WMSDs. The level of significance was set at *p* < 0.05 for these tests.

## Results

**Participants' details and the prevalence of WMSDs**: Table 1 displays the participants' details which comprise 91 (41.9%) males and 126 (58.1%) females; mostly married (46.5%) and within 51–60 years (28.6%). One hundred and forty-four (66.4%) were educated up to the tertiary level, and 128 (59.0%) employees worked between 5–8 hours. The majority of these participants (30.4%) had spent equal to/more than 16 years at work, and 30.5% were the most staff from a HEI. This table further reveals a high prevalence (71.9%) of WMSDs in this population. This prevalence rate denotes the percentage of participants who indicated the presence of WMSDs in one or more body regions.

**Table 1:** Participants' details and the prevalence of WMSDs

Variable	Frequency (%)
Sex	
Male	91 (41.9)
Female	126 (58.1)
Age (years)	
21 – 30	46 (21.2)
31 – 40	56 (25.8)
41 – 50	53 (24.4)
51 – 60	62 (28.6)
Marital Status	
Single	37 (17.1)
Married	101 (46.5)
Widowed/Separated	79 (36.4)
Educational status	
Primary	7 (03.2)
Secondary	66 (30.4)
Tertiary	144 (66.4)
Working hours	
1 – 4	37 (17.0)
5 – 8	128 (59.0)
≥ 9	52 (24.0)
Working experience (years)	
1 – 5	45 (20.8)
6 – 10	50 (23.0)
11 – 15	56 (25.8)
≥ 16	66 (30.4)
Institution	
HEI 1	58 (26.7)
HEI 2	66 (30.5)
HEI 3	63 (29.0)
HEI 4	30 (13.8)
WMSDs	
Present	156 (71.9)
Absent	61 (28.1)
**Total**	217 (100)

**Figure 1:**
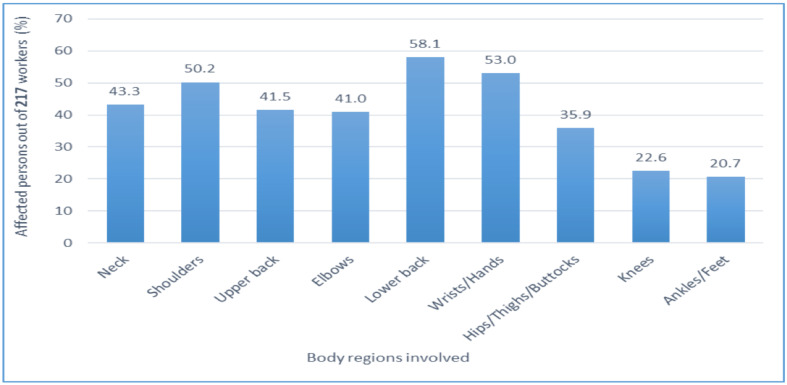
Body distribution of WMSDs amongst the affected participants

**Body distribution of WMSDs among the participants**: Figure 1 shows the distribution of WMSDs across the various body regions reported by the office workers. The lower back (58.1%), wrists/hands (53.0%) and shoulders (50.2%) were the most reported body regions affected by WMSDs. The hips/thighs/buttocks (35.9%), knees (22.6%) and ankles/feet (20.7%), all in the lower extremities, were the least indicated body regions.

**Association between prevalence of WMSDs and participants' details**: The association between the office workers' details and prevalence of WMSDs is presented in Table 2. A significant association existed between sex and prevalence of WMSDs (*p* = 0.004). Notably, 56 (61.5%) males reported the presence of WMSDs while 100 (79.4%) females made similar declarations. A significant association was also observed between advancing age and prevalence of WMSDs (*p* = 0.028). WMSD presence was higher (83.9%) in those aged 51–60 years and much lower (60.9%) in those aged 21–30 years. In contrast, there was not a significant association between prevalence of WMSDs and both marital (*p* = 0.069) and educational (*p* = 0.682) statuses of the employees respectively. Regarding work details, there was a significant association between this prevalence and increasing working hours (*p* = 0.003). More complaints of WMSDs were noted among many staff (76.9%) who worked for more than 9 hours compared to those (48.6%) who spent the least hours at work. There was not a significant association between prevalence of WMSDs among the participants and their institutions (*p* = 0.590). Furthermore, a significant association existed between this prevalence and increasing work experience (*p* = 0.014). WMSDs were mostly reported among personnel (80.3%) who had been working for 16 or more years than those (53.3%) with the least years.

**Table 2:** Association between the participants' details and prevalence of WMSDs

Variable	WMSDs		*X*^2^	*p*
	Present	Absent		
Sex			8.309	0.004*
Male	56 (61.5)	35 (38.5)		
Female	100 (79.4)	26 (20.6)		
Age (years)			9.107	0.028*
21 – 30	28 (60.9)	18 (39.1)		
31 – 40	36 (64.3)	20 (35.7)		
41 – 50	40 (75.5)	13 (24.5)		
51 – 60	52 (83.9)	10 (16.1)		
Marital Status			5.358	0.069
Single	32 (86.5)	05 (13.5)		
Married	72 (71.3)	29 (28.7)		
Widowed/Separated	52 (65.8)	27 (34.2)		
Educational status			0.764	0.682
Primary	06 (85.7)	01 (14.3)		
Secondary	48 (72.7)	18 (27.3)		
Tertiary	102 (70.8)	42 (29.2)		
Working hours			11.925	0.003*
1 – 4	18 (48.6)	19 (51.4)		
5 – 8	98 (76.6)	30 (23.4)		
≥ 9	40 (76.9)	12 (23.1)		
Working experience (years)			10.644	0.014*
1 – 5	24 (53.3)	21 (46.7)		
6 – 10	36 (72.0)	14 (28.0)		
11 – 15	43 (76.8)	13 (23.2)		
≥ 16	53 (80.3)	13 (19.7)		
Institution			1.915	0.590
HEI 1	42 (72.4)	16 (27.6)		
HEI 2	44 (66.7)	22 (33.3)		
HEI 3	46 (73.0)	17 (27.0)		
HEI 4	24 (80.0)	06 (20.0)		

Data are presented as frequency (%)

* Indicates statistical significance (*p* < 0.05)

**Relationship between reported risk factors and prevalence of WMSDs**: Presented in Table 3, Spearman's rank correlation showed that there were significant positive relationships (*p*<0.05) between the prevalence of WMSDs and the following reported risk factors: ‘awkward posture’, ‘sustained body position’, ‘improper bending’, ‘workplace stress’, ‘inappropriate furniture’ and ‘inadequate rest breaks’. Conversely, no significant relationship (*p* >0.05) was observed between the prevalence of WMSDs and other reported risk factors like ‘repetitive tasks’, ‘physical overexertion’, ‘multitasking’ and ‘neglecting precautions’.

**Table 3:** Correlation between the risk factors and prevalence of WMSDs

Risk factors	*r_s_*	*p*
Repetitive tasks	0.107	0.117
Awkward posture	0.349	0.000*
Sustained body position	0.372	0.000*
Improper bending	0.151	0.026*
Physical overexertion	0.113	0.097
Workplace stress	0.594	0.000*
Multitasking	0.092	0.175
Neglecting precautions	0.127	0.063
Inappropriate furniture	0.329	0.000*
Inadequate rest breaks	0.658	0.000*

* Indicates statistical significance (*p* < 0.05)

## Discussion

**Prevalence of WMSDs**: WMSDs were reported by most participants (71.9%) in this study, revealing a high prevalence rate. Comparable rates of 68.1% (15) and 69.6% (20) have been reported among Turkish office workers, with both depicting musculoskeletal symptoms which elicited difficulties whilst working and physical discomfort respectively. Similarly, other studies have supported our finding by stating high rates of 74% among Brazilians (16) and 80% in a Kuwaiti population (18).

Nigerian literature also affirms our finding among HEI office workers, with some authors reporting a 70% prevalence rate (26). Other authors uphold this finding in studies comprising such employees, but solely provided regional WMSD rates, stating highest rates which were above 70% (24,31). Another study presented a prevalence rate of 63% among similar personnel (25). There is evidence of poor ergonomic knowledge/practice among indigenous HEI staff (24,26) which might account for the raised occurrence of these disorders in our work setting. Overall, our finding reveals a high prevalence of WMSDs amongst office workers, which has also been identified in other research works (21,22).

**Body distribution of WMSDs**: The most reported body regions affected by WMSDs in our study were the lower back (58.1%), wrists/hands (53.0%) and shoulders (50.2%). Similar findings at the lower back (51.1%) and shoulders (49.2%) have been stated by some authors, though they reported that the neck was the most affected among bank office workers (18). Specifically, this finding is supported by reports of the lower back as the primary site for WMSDs, stating comparable rates of 61.3% (17) and 55.1% (15). Some indigenous research concurs with the above statement, but with higher rates of 74% (31) and 71.3% (22) at the lower back. Conversely, a lower back research among civil service office workers provided a lesser WMSD rate (23). Akodu et al. further supports our finding by revealing a similar WMSD rate of 48% at the shoulder. However, they also stated a much lesser rate at the wrist/hands (22). WMSD complaints (46.6%) at the wrists/hands reported by Labeodan et al. (26), affirm our finding.

The finding of our study may be attributed to some reports of unsatisfactory Nigerian HEI office/workstation settings, revealed to promote unnecessary physical efforts (27) and unhealthy postures (26). These reports also showed the lower back, wrist/hands and shoulders among the most reported body regions for WMSDs. Hence, prospective research is needed to verify this postulation, and importantly, review the current settings.

**Association with age**: Our study revealed a progressive increase in WMSDs, from the youngest to oldest population, as age displayed a significant association with their prevalence. This is consistent with previous reports among office personnel (18,19). Some authors, however, did not find such association between age and WMSD prevalence (13,20). A study also revealed that there was no such association, except for upper back symptoms, which were higher among young workers (14). Omokhodion and Sanya reported that though WMSDs was significantly higher in senior staff, it was doubtful that age had an influence as the prevalence did not increase with age (23). A British report in 2018 also supports our finding as it revealed significantly higher rates of WMSDs among older workers in diverse occupations. It further identified some age-related changes that affect functional abilities of adults with time, but highlighted that ageing does not inevitably bring illness/disease (32). Some older office workers might encounter challenges due to the indicated changes, which could lead to an increase in WMSDs.

**Association with sex**: The findings of this study demonstrated that women were more affected by WMSDs than men. Several authors concur with this observation of a greater WMSD prevalence amongst female office workers (14,15,17,18). Ardahan and Simsek also agreed that WMSDs were significantly higher among women; however, they noted that being male posed a risk of attaining these disorders in some body parts (20). Gender differences have been reported in indigenous studies on WMSDs, and these showed a higher prevalence of pain among men (23,26). Even so, other literature on such differences support our finding by revealing higher rates of musculoskeletal disorders among women (33,34). It has been suggested that distinctions in physiology and anthropometry in women might make them more vulnerable than men (35). Women also have the full responsibility of managing the home in Nigerian culture hence, they could commence various household chores even after a hectic day at the office; possibly eliciting more physical strain which would promote a surge in WMSDs.

**Association with working hours**: Office workers who spent more time at work, particularly above 8 hours per day (overtime), significantly reported more WMSDs in our study. This concurs with reports of increased WMSDs among such personnel who spent more time per day working (21), especially on a computer (20). Celik et al. found out that time spent at work was associated with WMSDs, but this exclusively depicted the sitting duration which affected some body regions (15). Lee at al. also support our finding by revealing that long working hours increased WMSD prevalence, and suggested a possible influence of the prolonged exposure to the physical demands of work (36). Similarly, it was expounded that lengthy working hours can relatively decrease the time to relieve stress and recover from accumulated fatigue (37), which might harm the body and elicit WMSDs. This information plausibly applies to some HEI office staff who work for protracted hours.

**Association with work experience**: In our study, WMSD prevalence was observed to significantly increase amongst workers; from those with the least to the most work experience. Similar observations were made in personnel who had worked for more years in an office (18,27). Other reports agree with our finding and have specifically linked increased WMSD occurrence to the workers' years of computer usage (17,20). Remarkably, a high association between work experience and WMSD prevalence has been revealed in systematic review, providing such evidence but among distinct personnel (5). Our finding seems to demonstrate that more work experience may not necessarily include development in areas outside one's job description, especially towards the effective management of physical or mental demands of the job; which could help reduce the susceptibility to WMSDs. This stance calls for a better exploration among these office employees.

**Relationship with reported risk factors**: Awkward posture, sustained body position and improper bending had significant positive relationships with WMSD prevalence in this study. This is affirmed by a systematic review which provided evidence supporting a causal relationship between awkward posture and WMSDs (11). Some authors also reported that this faulty posture influenced the musculoskeletal pain felt by office workers (15). They also noted that sustained body position had a significant effect on WMSD occurrence, as working in the same position for a long time may put the muscles under stress, reduce blood flow, lead to fatigue and ultimately, elicit pain/tissue damage (38). Improper bending which denotes a swift incorrect movement of a body part to instantly achieve a goal like reaching for computer monitors, files in cabinets or items on the floor, could cause discomfort and strain. This action might lead to the adoption of awkward postures, and if the body is sustained in this faulty position, it experiences deleterious effects which have been reported to contribute to WMSD prevalence (16,35,39).

Our study also revealed that workplace stress, inappropriate furniture and inadequate rest breaks had such significant relationships with WMSD prevalence. Accordingly, moderate or extreme levels of mental stress in the workplace have been reported to influence the musculoskeletal disorders experienced by office workers (15,35), as these can augment physical stress and lead to superfluous exertion whilst performing office tasks (40). Other authors have also affirmed this adverse influence on WMSD prevalence among HEI employees (25). There is evidence of an association between poorly suited chairs/desks and WMSDs (39), which supports our finding. Some authors have reported this negative association of inappropriate furniture and WMSDs among office workers (15). Indigenous research further revealed these workers' dissatisfaction with substandard seats (27) and their preference of proper sized desks with adjustable chairs which had back support (21). Additionally, working without adequate rest breaks has been reported to influence WMSD prevalence (15,17,20,35). Though these reports cited several breaks taken at different times and for varied durations, they unanimously explicated the invaluable relief from mental and physical stress attained by taking a satisfactory break from work activity, to check the development of WMSDs.

**Implications, limitations and future studies**: Our findings highlight the need to resolve the high WMSD prevalence among office workers via proper screening and intervention. Specific attention should be given to workers who are older, female, more experienced and work for long hours, as such populations display higher risks for these disorders. Regular observation of each worker is vital towards managing WMSDs, not depending on their awareness of these disorders or possible modern office settings, as risk factors can be detected and curtailed. Ergonomics education of office workers may be insufficient to address WMSDs hence, we suggest the provision of ideal workstation components which ought to be tailored to each worker and regularly serviced, as well as the implementation of policies targeted at improving employees' health. Workers should be actively involved in executing and maintaining these measures, to successfully inhibit WMSDs.

The use of a self-report questionnaire is prone to response bias, which provides a probability of influencing the finding of this study. Causal inferences cannot be made due to this study's design. Future studies should consider longitudinal and experimental study designs to provide deeper insights on WMSDs and evaluate interventions to check these disorders.

## Conclusion

WMSDs currently pose challenges to office workers, so this questionnaire-based study was executed to explore such disorders among those working in HEIs. A high prevalence was found in this population whilst the lower back, wrist/hands and shoulders were revealed as the most commonly affected body regions. Personnel who were older, female, more experienced and work for longer hours, exhibited a high predisposition to these disorders. Several risk factors reported by these HEI employees were shown to substantially influence WMSD prevalence, highlighting the need for their successful detection and curtailment.
